# Evaluation of dyssynchrony in children with dilated cardiomyopathy: a comparison of electrical and mechanical delay using Doppler, tissue imaging and strain

**DOI:** 10.1186/s43044-025-00633-3

**Published:** 2025-04-16

**Authors:** Fariba Rashidi Ghader, Mohammad Mahdavi, Hossein Mehrali, Mohammad Dalili, Hossein Shahzadi, Reza Abbaszade

**Affiliations:** Department of Pediatric Cardiology, School of Medicine, Rajaie Cardiovascular Research and Medical Institute, Iran University of Medical Sciences, Tehran, Iran

**Keywords:** Cardiomyopathy, Dilated, Conduction defect

## Abstract

**Background:**

Dilated cardiomyopathy (DCM) is a primary myocardial disease characterized by systolic dysfunction, which can lead to disparity and disorganized contraction, commonly referred to as dyssynchrony. Three types of dyssynchrony include atrioventricular (AVD), interventricular (inter-VD), and intra-LV dyssynchrony (intra-VD). We aimed to investigate the prevalence and interdependence of electrical and mechanical dyssynchrony in order to elucidate the optimizing patients for cardiac resynchronization therapy (CRT).

**Results:**

A total of 37 DCM patients (1–17 years, 51% female) were included in this cross-sectional study. Regarding the intra-VD, inter-VD, and AVD, the study showed inter-VD in 37%, 27%, and 48% by Doppler, Doppler tissue imaging (DTI), and color-coded DTI methods, respectively; however, 70% showed right ventricular free wall delay based on the presence of peak of strain after pulmonic valve closure. 86.5% (32/37) of patients show intra-VD. 100% (8/8) of DCM patients with prolonged QRS (QRSc ≥ 120 ms) had intra-VD, of which 12.5% (1/8) had mild, 25% (2/8) mod, and 62.5% (5/8) severe dyssynchrony. However, 82% (24/29) of patients with narrow QRS (QRSc < 120 ms) also had intra-VD, of which 17% (3/24) were mild, 62.5% (15/24) mod, and 25% (6/24) severe. There were 57% (21/37) of patients with AVD. 77% (10/13) of DCM patients with prolonged PRc (PR ≥ 200 ms) had AVD of which 31% (4/13) of patients had mild, 31% (4/13) mod, and 15% (2/13) severe AVD, while among PRc < 200 ms 46% (11/24) had AVD, of which 37.5% (9/24) had mild AVD, 4% (1/24) mod, and another 4% severe AVD. LVEF was lower and LV GLS, mortality, Pro-BNP, NYHA FC, and severity of intra-VD were higher in the group with QRS ≥ 120 ms, and PR ≥ 200 ms. 27% of patients were expired during the year of study. There was a significant direct correlation between mortality rate, NYHA FC, and pro-BNP with the severity of intra-, inter-VD, and AVD. The most delayed horizontal segments were inferolateral, anterolateral, anterior, and anteroseptal sequentially, while the highest level of vertical dyssynchrony (base to apex) was observed in inferoseptal, inferolateral, and anteroseptal walls in order.

**Conclusions:**

Our findings indicated that DCM causes both intra- and inter-VD, associated with QRS duration concerning severity, which also results in AVD that are correlated with PRc interval. Notably, a substantial proportion of patients with narrow QRSc also demonstrated intra-VD and inter-VD, while nearly half of those with normal PRc exhibited AVD. Collectively, these observations suggest a lack of complete correspondence between electrical and mechanical dyssynchrony.

## Background

Dilated cardiomyopathy (DCM) is a primary, progressive and usually irreversible myocardial disorder characterized by left ventricular (LV) or biventricular dilation and global systolic dysfunction in the absence of structural heart disease such as congenital heart disease (CHD), valvular disorders, hypertension, or coronary artery disease [1]. The extent of ventricular dysfunction has traditionally been assessed by M-mode, Doppler, and tissue Doppler imaging (TDI) [2, 3]. More recently, deformation studies have provided additional insights [2, 3]. In a healthy heart, myocardial segments exhibit highly synchronized onset and peak motion across all planes. The activation of LV is disturbed in DCM, leading to a delayed activation of the lateral wall. When the earlier activated interventricular septum contracts and produces very little mechanical work pushing blood simply toward the relaxed lateral wall, then the late-activated LV lateral wall is pre-stretched so it performs a higher workload. This disparity, known as dyssynchrony, results in different expression of multiple proteins, heterogeneous calcium-handling properties, changes in wall thickness, and variations in electrical conduction speeds within the LV [4]. Consequently, dyssynchrony disorganizes cardiac contraction and reduces pumping efficiency. Patients with severe LV systolic dysfunction and prolonged QRS interval have been reported to have higher morbidity and mortality rates than those with normal QRS duration [5].

Dyssynchrony can be categorized into three types: atrioventricular dyssynchrony (AVD), characterized by delayed ventricular contraction; interventricular dyssynchrony (inter-VD), exhibiting discordance between LV and right ventricular (RV) contractions; and intra-LV dyssynchrony (intra-VD), marked by discordant contractions of different LV walls [5]. Cardiac resynchronization therapy (CRT) has been demonstrated to reduce heart failure symptoms and hospitalizations, enhance quality of life, and improve exercise capacity and overall prognosis [5]. CRT eliminates the mechanical dyssynchrony, thereby improving systolic and diastolic dysfunction, as well as functional mitral regurgitation, in patients with DCM [5]. Historically, many studies have employed the presence of electrical dyssynchrony (QRS ≥ 120 ms) as an inclusion criterion [6]. Nonetheless, some patients with wide QRS fail to respond adequately to CRT, potentially due to the absence of mechanical dyssynchrony. Conversely, certain patients without wide QRS exhibit better responses, which can be attributed to the lack of a strong correlation between electrical and mechanical dyssynchrony [7].

There are several reasons to measure mechanical dyssynchrony in addition to electrical dyssynchrony. Firstly, QRS duration alone does not provide detailed information on the activation timing of different LV regions and fails to distinguish between LV and RV activation [4, 6]. Secondly, coordinated myocyte contraction is a complex process essential for moving blood through the aortic and pulmonic valves. This process can encounter delays in electromechanical coupling, which is initiated by electrical activity but only represents an early step [4, 6]. Thirdly, mechanical dyssynchrony may hold more significance in optimizing patient selection for CRT and predicting cardiovascular events in heart failure patients [4, 6].

### Objectives

Various methods have been employed to detect mechanical dyssynchrony, including cardiovascular magnetic resonance (CMR), radionuclide scintigraphy, invasive conductance catheter recordings, and echocardiography [7]. Echocardiography is widely recognized as a useful, feasible, and cost-effective tool to identify motional disparities using velocity, displacement, or strain measurements [5, 6, 8]. In this study, our objectives were to evaluate the prevalence of different types of mechanical dyssynchrony in patients with DCM, examine potential relationships between PR interval and AVD, as well as between QRS duration and inter-VD and intra-VD, and provide an overview of the most commonly employed echocardiographic methods to obtain mechanical dyssynchrony in order to elucidate the optimizing patients for CRT.

## Methods

### Study design

DCM patients with LVEF < 50% of the age limit of 6 month–17 years were enrolled in the study as outpatients and inpatients. This single-center cross-sectional study was conducted throughout 1 year at our institute. The patients with CHD or arrhythmia were excluded. All patients underwent evaluation for electrical and mechanical dyssynchrony using ECG and echocardiography, respectively. Demographic data including age, body surface area (BSA), sex, and some laboratory tests included NT pro-BNP, bilirubin, SGOT, SGPT, creatinine, and hemoglobin were collected for analysis. New York Heart Association functional class (NYHA FC) was determined based on patient and parent reports.

### Electrocardiography

Electrocardiography was done at about the same time as echocardiography in relaxing and calming state, by standardized 12 leads at voltage calibrated so that 1 mV = 10 mm and speed of 25 mm/s. Then ECG interpreted as QRS duration and PR interval were measured and adjusted according to bazett’s formula (QRS dur/√RR). Left bundle branch block (LBBB) was identified by the presence of QRS > 120 ms in leads V_1_–V_6_ with notching or slurring, poor R/S progression, notching in leads I and AVL, and/or left axis deviation. Participants were then divided into two groups based on QRS duration (≥ 120 ms and < 120 ms) and PR interval (≥ 200 ms and < 200 ms).

### Echocardiography

**Fig. 1:**
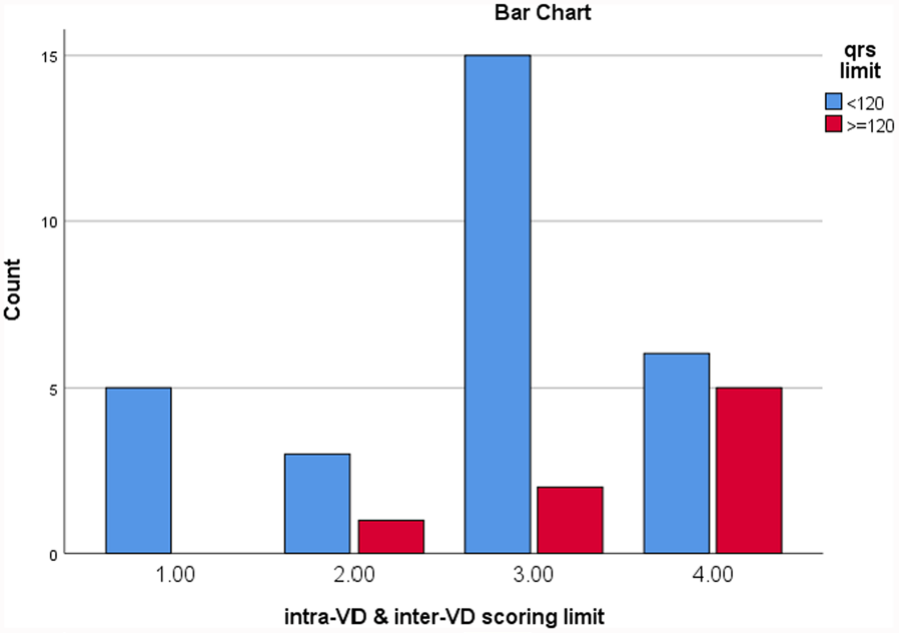
Severity of dyssynchrony scoring 1–4 from no VD to severe VD, score 1 = no dyssynchrony: It means that, the longer the QRS interval, the more severe the VD. Refer to list of abbreviations before references

**Fig. 2:**
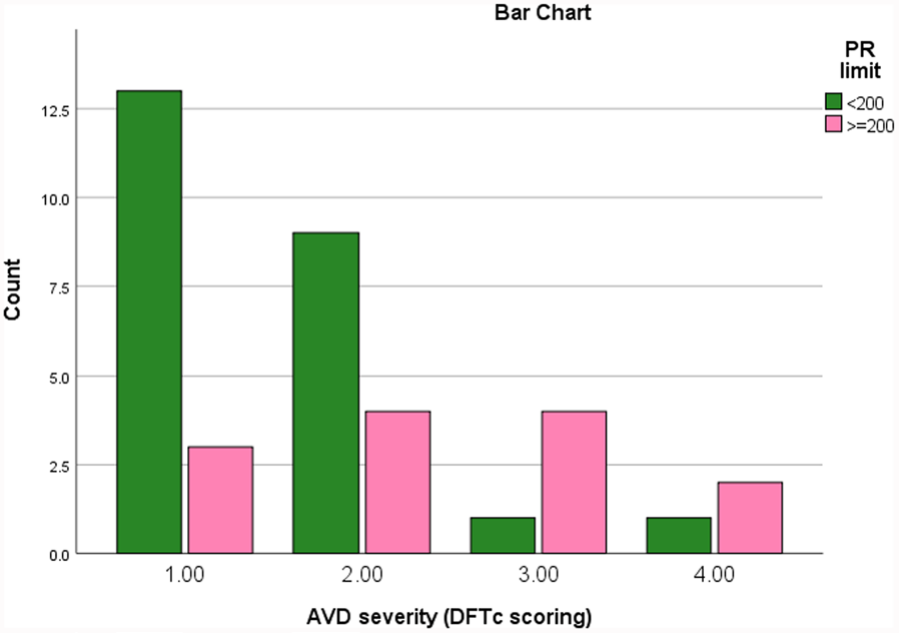
Severity of AV dyssynchrony scoring 1–4 from no AVD through severe AVD (score 1 = no AVD, 2: 0.35 < DFT ≤ 0.40, 3: 0.3 < DFT ≤ 0.35, 4: DFT ≤ 0.3; it means that: The longer the PR interval, the more severe the AVD. Refer to list of abbreviations before references

Echocardiographic examinations were performed using Doppler, TDI, color-coded tissue, color-coded M-mode, and 2D techniques. Ventricular volumes and EF were determined using the Simpson biplane method on two available echocardiography systems: the GE s60 (General Electric, Norway), primarily used for inpatients with NYHA FC ≥ 2 in the heart failure ICU ward, and the Philips iE33 for other patients. An experienced pediatric echocardiographer conducted all examinations. High-quality two-dimensional (2D), color-coded TDI, and pulsed-wave (PW) TDI images were acquired from the apical 2-, 3-, and 4-chamber views. Color-coded TDI was used to sample regions of interest (ROI) in six basal and six mid-segments. The speckle-tracking analysis also was performed on acceptable quality acquisited 2D images to obtain global longitudinal strain (GLS), global circumferential strain (GCS), and time-to-peak strain (Figs. 1 and 2).

**Fig. 3:**
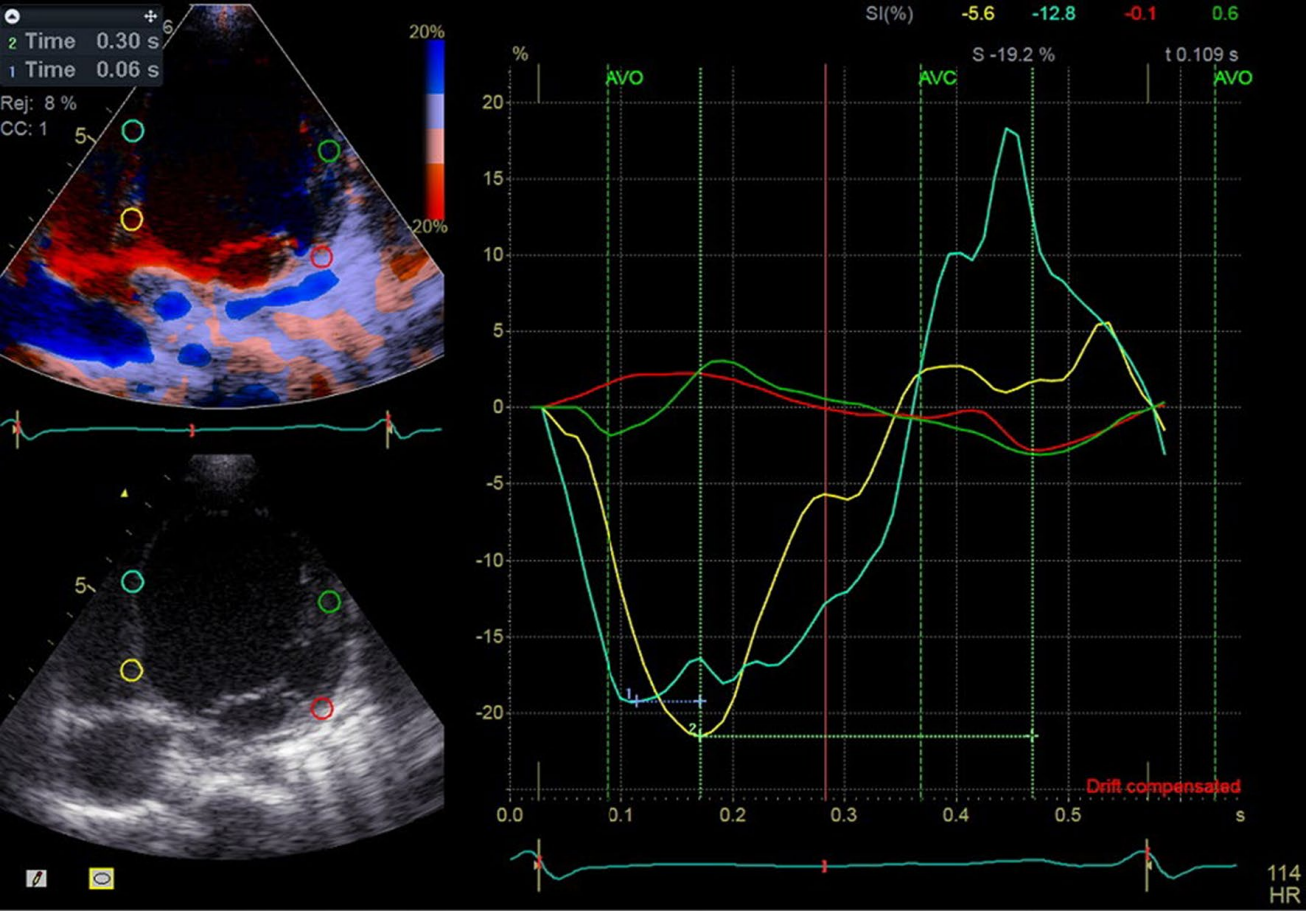
Electrosystolic delay of mid (blue and green curves) and basal portion (yellow and red curves) of inferoseptal and anterolateral walls as demonstrated by strain analysis, note the systolic delay of base and mid portion of anterolat wall as peak of strain appears after aortic valve closure (AVC) and early stretching at first third of systole and early peak of strain of base and mid of inferoseptal at first third of systole instead of end of systole (classical pattern), notice the time difference of peak of strains of opposing segments, Time 1 = 0.06 s (difference between basal and mid portion of IS),Time 2 = 0.30 s (difference between basal of IS and AL)

**Fig. 4:**
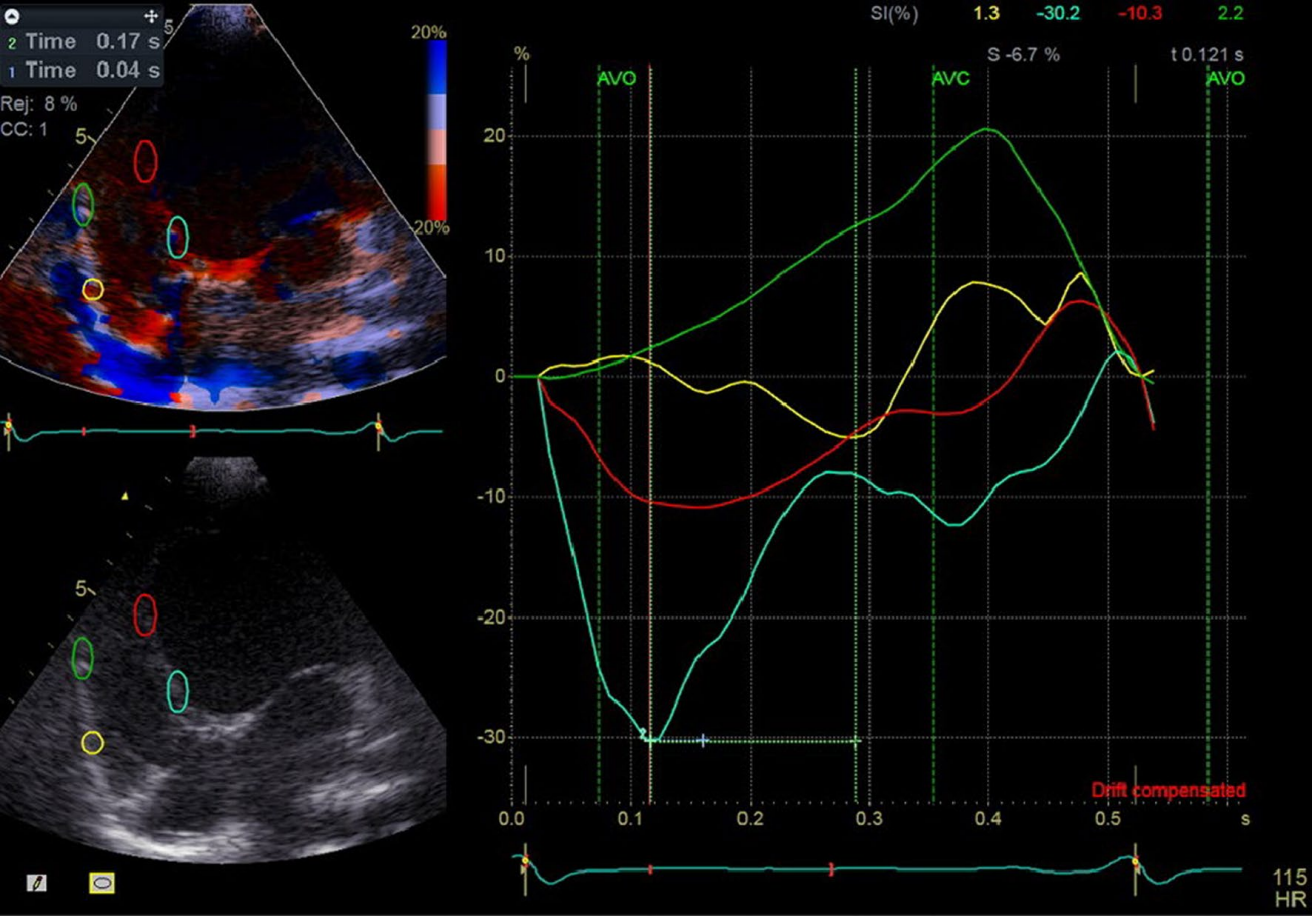
Electrosystolic delay of mid (red and green curves) and basal portion (yellow and blue curves) of inferoseptal and RV free walls as demonstrated by strain analysis, note the paradoxical motion of mid-portion of RV free wall where peak of strain appears at third portion of diastole, normal appearance of peak of strain of basal of RV free wall (yellow curve), early peak of strain of basal and mid of IS at first third of systole instead of end of systole, notice the time difference of peak of strains of opposing segments, Time 1 = 0.04 s (difference between basal and mid portion of IS),Time 2 = 0.17 s (difference between basal of IS and RV free wall)

**Fig. 5:**
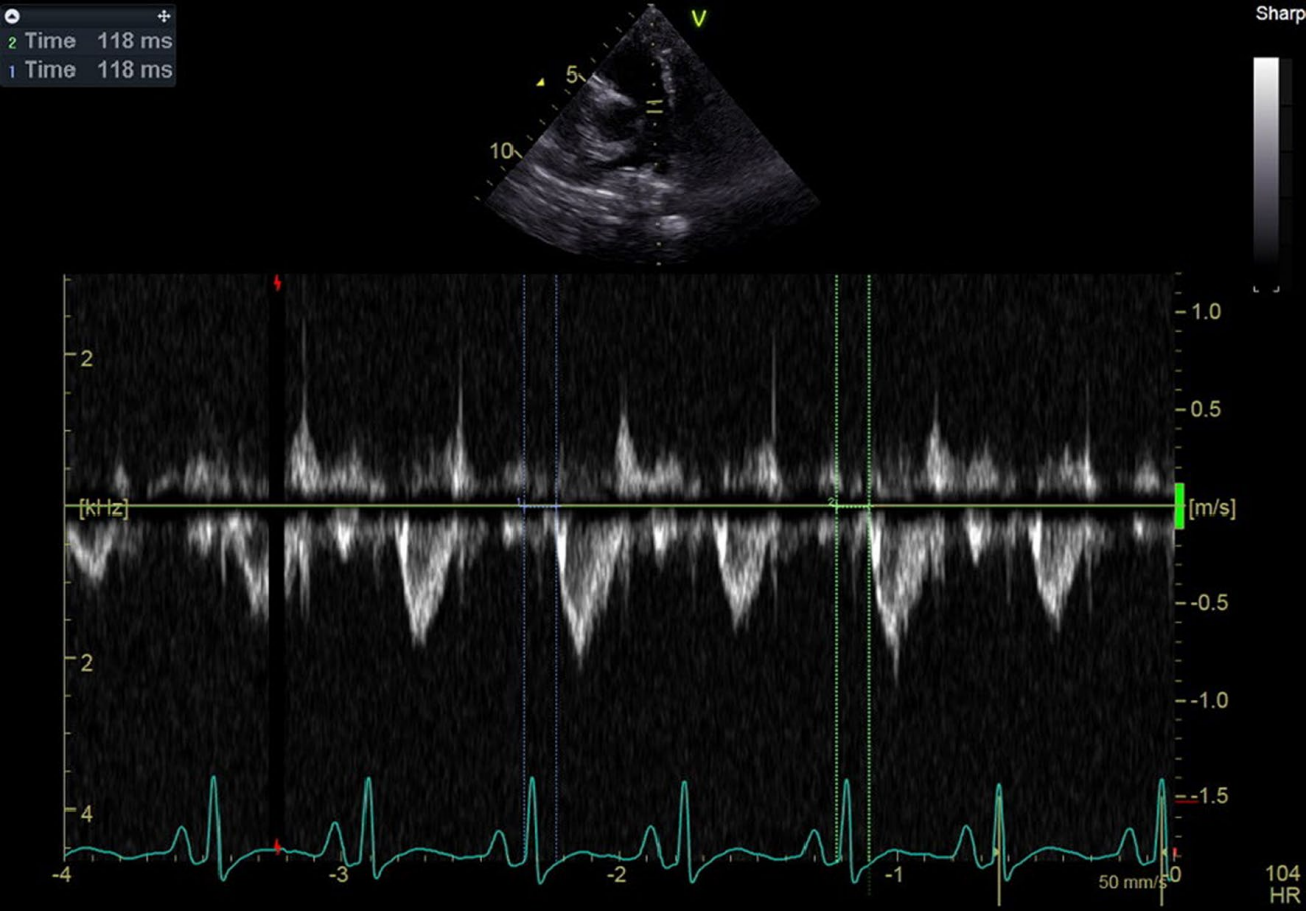
Electromechanical delay of RV by Doppler analysis = 118 ms

**Fig. 6:**
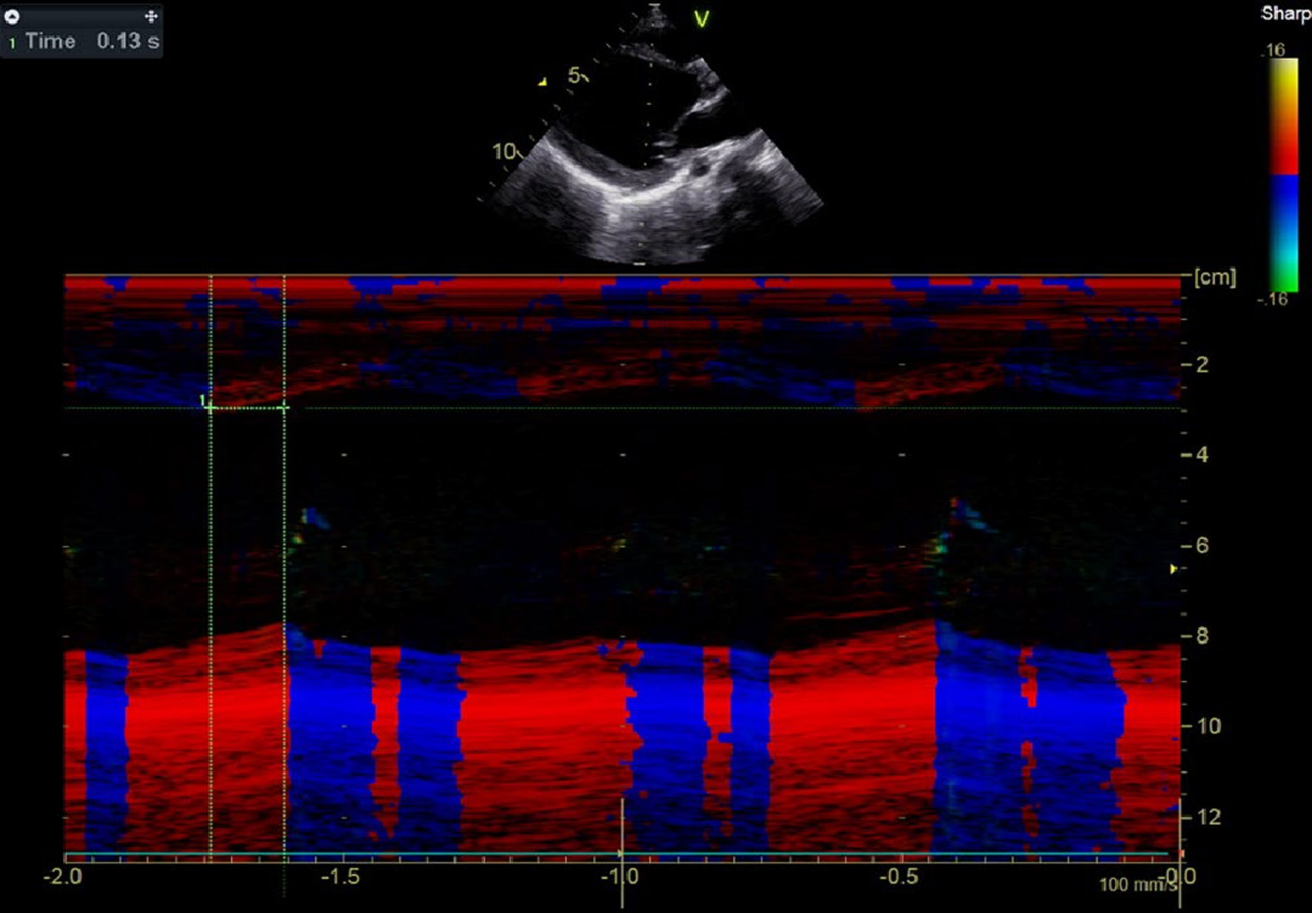
Time difference of septum and posterior wall of LV (SPWMD) = 0.13 s

**Fig. 7:**
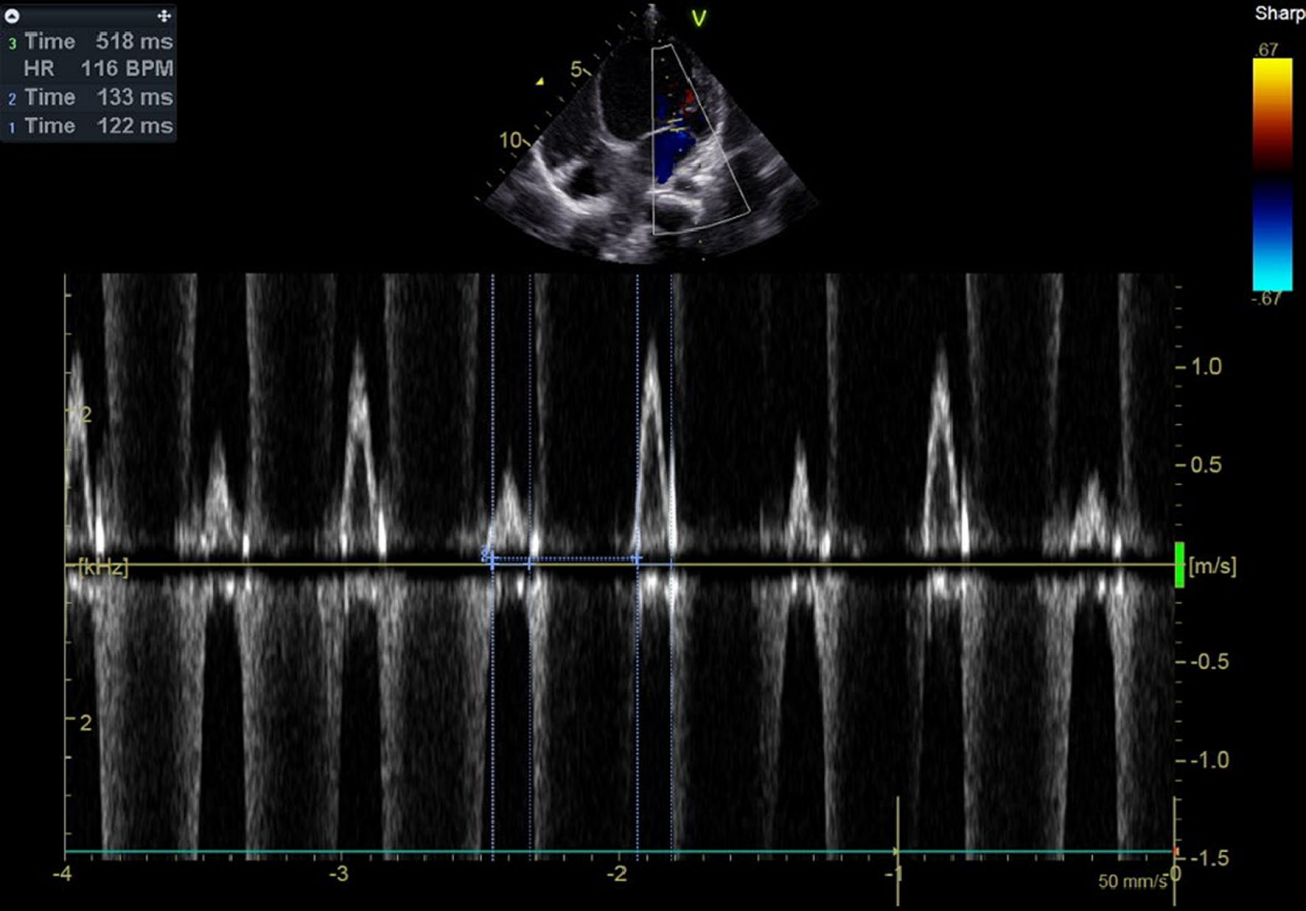
Diastolic filling time = 133, 122 ms (avge = 127 ms), RR interval = 518 ms, DFTc = 127/518 = 25%), note prolonged DFTc < 40% in DCM

**Fig. 8:**
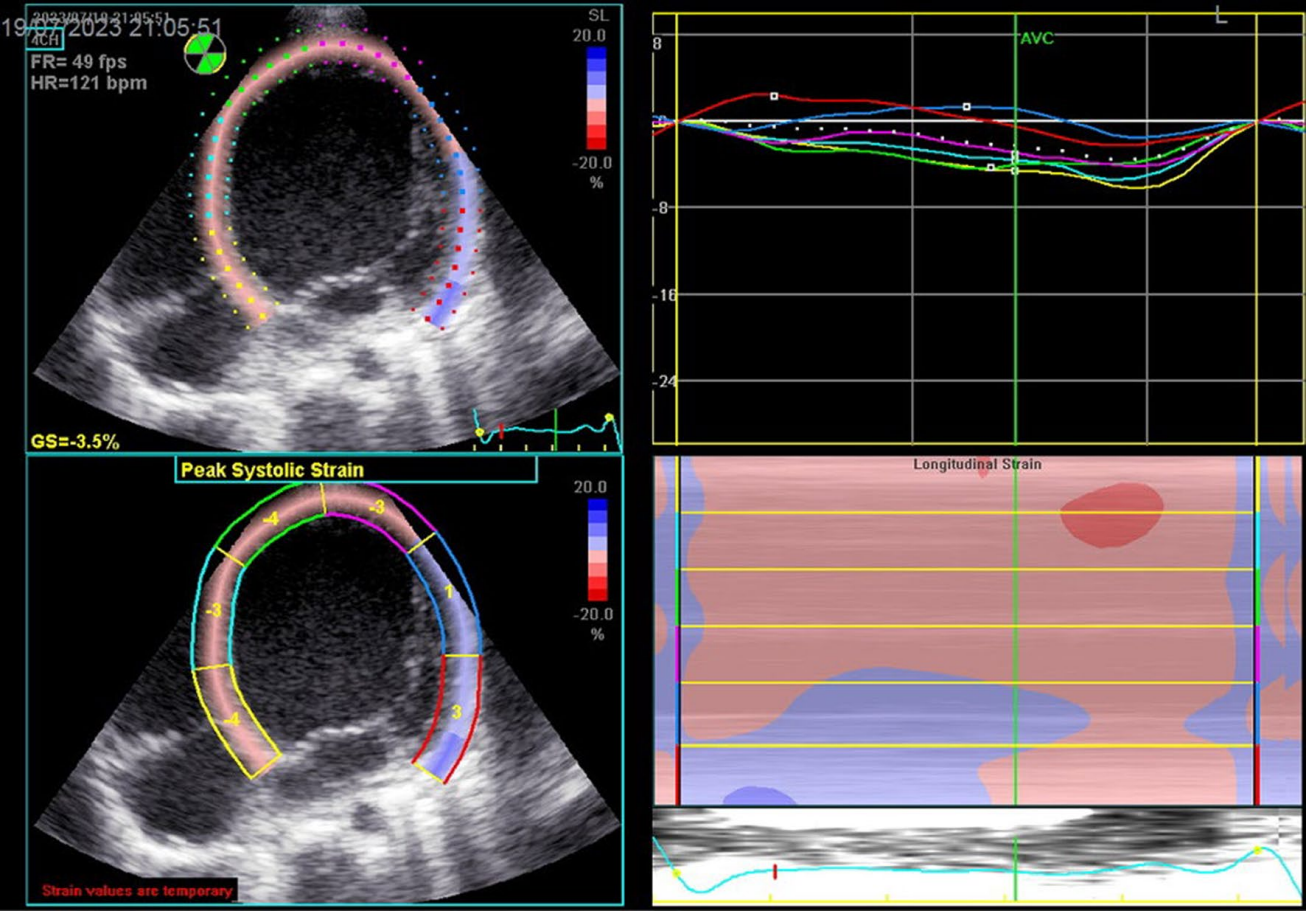
Decreased GLS of LV at 4c view = − 3.5%, note the delayed appearance of peak of systolic strain after aortic valve closure (AVC) of most segments

For the calculation of electromechanical delay and electrosystolic delay, the time interval from the beginning of the QRS wave to the onset of aortic and pulmonary valve opening was utilized. These values were derived from PW TDI, Doppler, and color-coded TDI. Electrosystolic delay was calculated using the time interval from the beginning of the QRS wave to the aortic and pulmonary valve peak systolic velocity. Additionally, the time-to-peak longitudinal strain obtained from speckle-tracking analysis and the septal-to-posterior wall motion delay (SPWMD) on color-coded M-mode was employed to assess dyssynchrony. All the measured parameters except speckle-tracking analysis were calculated as the average of three consecutive beats (Figs. 3, 4, 5, 6, 7 and 8).

According to QRS duration and PR interval, the patients were divided into two subgroups: for QRS duration group 1 with QRS duration ≥ 120 ms and group 2 with a narrow QRS < 120 ms. And for PR interval further two groups included PR interval ≥ 200 ms and PR interval < 200 ms, and then the groups compared in terms of mechanical dyssynchrony parameters.

For the assessment of AVD, a mean left ventricular filling time (LVFT)-to-cardiac cycle ratio (LVFT/RR × 100) below 40% was considered abnormal. AVD grading was determined by measuring diastolic filling time (DFT) as follows:

1. Grade I: No AVD
2. Grade II: 0.35 s < DFT ≤ 0.40 s
3. Grade III: 0.3 s < DFT ≤ 0.35 s
4. Grade IV: DFT ≤ 0.3 s

To evaluate inter-VD: The difference between the two values as an inter-VD > 40 ms was considered for the cutoff value of inter-VD; however, it was adjusted by patients’ heart rate (HR) by the formula of inter-dyssynchrony × 75/HR [9]. Inter-VD was determined based on the difference of opening of PA and AO at Doppler, spectral tissue Doppler imaging (DTI), and peak of RV free wall and septal Sm at color-coded DTI (cc DTI).

We determined the electrosystolic delay (ESD) by PW DTI from the onset of the QRS complex to the positive peak systolic velocity (Sm) for four basal segments (septal, lateral, inferior, and anterior).

Intra-LVD was identified when absolute differences between two opposing segments exceeded 65 ms. Intra-LVD was also determined using strain analysis of the following six basal and six mid-segments: inferoseptal, anterolateral, inferior, anterior, anteroseptal, and inferolateral [7]. Then the time interval from the onset of the QRS complex to the peak of strain was measured. Segments with the peak of strain occurring after aortic and pulmonic valve closure were identified as delayed mechanical segments in LV and RV, respectively. These delayed segments were further classified into three groups based on the degree of dyssynchrony:

1. *Mild dyssynchrony*: Peak strain appears in the first third of diastole or is delayed until the second third after aortic valve closure.
2. *Moderate dyssynchrony*: Peak strain occurs between the first and second third of diastole.
3. *Severe/paradoxical dyssynchrony*: Peak strain appears in the last third of diastole or is paradoxically positive.

A scoring system was used to quantify dyssynchrony at different segments, taking into account the degree of dyssynchrony as follows: the number of segments with mild dyssynchrony + 2 × (the number of segments with mod dyssynchrony) + 4 × (the number of segments with severe dyssynchrony or paradoxical motion).

The total severity score was then recalculated and categorized as absent (no segments with dyssynchrony), near absent (only 1 or 2 segments with peak strain appearance at less than one-third of diastole), presence of dyssynchrony as depicted in Table 6.

### Statistical analysis

Data were collected and analyzed with SPSS 26. Continuous and qualitative variables were presented as mean or median ± standard deviation. Comparisons between frequencies in the two groups were performed using crosstab with the Chi-square test. Correlations between electrocardiographic, echocardiographic, and demographic parameters were explored using Pearson’s or spearman’s correlation. To check the correlation of electromechanical variables, *P* value derived from χ^2^ test and odds ratio (OR) with CI = 0.95 for ordinal variables; additionally, *P* value derived from Mann–Whitney and effect size for quantitative variables were applied.

## Results

A total of 37 patients with the diagnosis of DCM were enrolled in the study. Their characteristics are presented in Table 1, as demonstrated the mean age of DCM patients was 9.4 ± 4.53 with the range of 1–17 year consisted of 18 male and 19 female 27% of whom were expired during the year of study. Echocardiographic and electrocardiographic parameters are demonstrated in Tables 2 and 3, respectively. The fibrosis based on as assessed by late gadolinium enhancement (LGE) and non-compaction LV (NCLV) based on CMR and echocardiographic methods were also reported. 6 out of 37 patients who were younger and with milder intensity DCM had not done cardiac MRI, which their EF and volumetric data were obtained using biplane Simpson’s echo method.

**Table 1:** Patients’ characteristics

	Mean ± SD, unit; median ± SD	Unit	Numbers/percent; min/max
Mortality			10, 27%
Transplant			6, 16%
Age	9.4 ± 4.53	Year	1–17
Gender	M/F = 0.95		18 Male/19 female
SA	1.07 ± 0.41	M^2^	
NT pro-BNP	8360 ± 10,107	Pg/ml	20–35,000
Creatinine	0.73 ± 0.31	Mg/dl	
SGOT	45 ± 41	Unit/l	
SGPT	42 ± 111	Unit/l	
Bil	1.27 ± 1.01	Mg/dl	
Hb	11.7 ± 1.52	g/dl	
NYHA FC	3 ± 0.93 (median)		FC 1–4

Refer to list of abbreviations before references

**Table 2:** Patients echocardiographic parameters

	Mean ± SD; median ± SD	Unit	Numbers/percent; min/max
FAC	0.27 ± 0.07	–	
RV Sm	8.6 ± 2.35	Cm/s	
MR degree	3 ± 0.72		
TR degree	2 ± 0.64		
LV EDV	183.1 ± 61.8	Ml	
LV ESV	151.6 ± 63.7	Ml	
RV EDV	121.6 ± 51.3	Ml	
RV ESV	87.2 ± 45.1	Ml	
LVEF	20% ± 0.12		7–50%
RVEF	29% ± 0.16		
NCLV	0 ± 0.95		No: 23(62%)
			Mild: 8(21%)
			Mod: 3(8%)
			Severe: 3(8%)
LGE	0 ± 0.98		No: 16(43%)
			Mild: 7(19%)
			Mod: 7(19%)
			Severe: 2(5%)
Clot	0 ± 0.77		No: 31(83%)
			Small: 3(8%)
			Mod: 1(2%)
			Large: 2(5%)
GLS LV	− 0.07 ± 0.048		0.0 to (− 0.18)
GCS LV	− 0.10 ± 0.054		− 0.02 to (− 0.20)
GLS RV	−.0099 ± 0.054		− 0.02 to (− 0.20)

Refer to list of abbreviations before references

**Table 3:** Patients ECG parameters

	Mean ± SD; median ± SD	Unit	Numbers/percent; min/max
QRSc duration	101.4 ± 28.4	Ms	
QRSc ≥ 120 ms			QRSc ≥ 120 ms = 8 (21.6%)
QRSc < 120 ms			QRSc < 120 ms = 29 (78.4%)
PRc interval	191.24 ± 40.4	Ms	
PRc ≥ 200 ms			PRc ≥ 200 ms = 13 (35.1%)
PRc < 200 ms			PRc < 200 ms = 24 (64.9%)
LBBB	1 ± 0.31	No = 1,yes = 2	
Yes			Yes: 4 (10.8%)
No			No: 33 (89.2%)

Refer to list of abbreviations before references, QRSc, PRc: corrected by Bazette’s formula

Data on dyssynchrony, including inter-VD, intra-VD, and AVD, are presented in Table 4. Further details on inter-VD and intra-VD obtained using Doppler, TDI, and color-coded TDI methods are shown in Table 5. Information regarding RV free wall delay based on the presence of peak strain after pulmonic valve closure and the classification of severity can be found in Table 6.

**Table 4:** Patients dyssynchrony parameters

	Mean ± SD; median ± SD	Unit	Numbers/percent; min/max
Horizontal dyssynchrony		Ms	
2c(ant-inf difference)	124.5 ± 40.4		20–200
3c(AS-IL difference)	151.6 ± 50.1		60–300
4c(IS-AL difference)	142.3 ± 55.1		30–300
Vertical dyssynchrony		Ms	
Ant	93 ± 41.1		35–190
Inf	81.9 ± 55.3		10–250
AS	92.3 ± 40		20–170
IL	99.7 ± 64.6		10–350
IS	103.9 ± 56.9		20–270
AL	77.7 ± 47.6		10–200
Horizontal LV-RV dyssynchrony	105.5 ± 39.1	Ms	20–180
Vertical RV free wall dyssynchrony	75 ± 62.6	Ms	10–270
AVD	40% ± 10%		22–65%
SPWMDc	102.5 ± 52.4	Ms	25–220

Refer to list of abbreviations before references. To evaluate horizontal dyssynchrony, compare base to base and mid to mid at each standard views of 2c, 3c, and 4c views, then obtaining mean value

**Table 5:** Patients’ inter-VD and intra-VD parameters by Doppler, Doppler DTI, color-coded DTI and M-mode

	Mean ± SD; median ± SD	Unit	Numbers, percent; min/max	Inter-VD Number/percent
EMD LV dop	107.3 ± 18.2	Ms	83–174	
EMD LV dop DTI	94.7 ± 21.1	Ms	40–167	
EMD LV CC DTI	81 ± 36.7	Ms	14–177	
ESD LV dop	188.3 ± 35.3	Ms	116–280	
ESD LV dop DTI	172.6 ± 30.7	Ms	118–255	
ESD LV CC DTI	180.5 ± 58.6	Ms	55–355	
EMD RV dop	91.1 ± 20.1	Ms	60–140	
EMD RV dop DTI	81.7 ± 16.1	Ms	50–134	
EMD RV CC DTI	58.6 ± 48.6	Ms	8–240	
ESD RV dop	162 ± 33.3	Ms	92–250	
ESD RV dop DTI	155.1 ± 39	Ms	100–270	
ESD RV CC DTI	140.4 ± 73.6	Ms	26–350	
AO-PA opening dop diff	30.7 ± 10.9	Ms	15–48	14/37 (37%)
AO-PA opening dop DTI diff	26.1 ± 12.7	Ms	8–48	10/37 (27%)
AO-PA opening cc DTI diff	35.9 ± 17.2	Ms	5–68	18/37 (48%)
AO-PA peak dop diff	29.4 ± 27.3	Ms	− 17 to 82	10/37(35%)
AO-PA peak dop DTI diff	20 ± 2	Ms	− 52 to 70	9/37(24%)
AO-PA peak cc DTI diff	40 ± 39.7	Ms	− 60 to 118	11/37(29%)
SLWSDc DTI	38 ± 11.2	Ms	12–50	9/37(24%)
SLWSDc CC	54.2 ± 32.3	Ms	30–101	12/37(32%)
SPWMD	102.5 ± 52.4	Ms	25–220	14/37(37%)

Refer to list of abbreviations before references

**EMD**: Electromechanical delay: Time difference from the onset of QRS to the AO valve opening in LV and PA opening in RV which have been acquired by Doppler (method 1), DTI (method 2) and color-coded DTI (method 3)

**ESD**: Electrosystolic delay: Time difference from the onset of QRS to the peak of aortic flow wave in LV and PA flow wave in RV which have been obtained by Doppler (method 1) and DTI (method 2) and color-coded DTI (method 3)

**AO-PA difference** has been calculated by subtracting the EMDs and ESDs of LV and RV acquired by all three methods

**SLWSDc:** Septo lateral wall systolic (corrected by HR) delay by color-coded

**Table 6:** Classification of dyssynchrony severity

Dyssynchrony	Number/percent of subjects intra-VD	SLWSD DTIc ≥ 65 ms	SLWSDcc c ≥ 65 ms	Number/percent of subjects free wall of RV	Total
Absent (or near absent): score = 0–2	5, 13.5%	29, 78%	25, 67.5%	11, 30%	LV = 5 RV = 11
*Present*					LV = 32 RV = 26
Mild (2 < score ≤ 10)	4, 10.8%	1, 2.7%	2, 5%	26, 70%	
Mod (10 < score ≤ 20)	17, 46%	3, 8%	4, 10.8%	0	
Severe/pdx (score > 20)	11, 29.7%	5, 13.5%	6, 16%	0	

Refer to list of abbreviations before references

ECG parameters were divided into two groups as QRSc ≥ 120 ms and QRSc < 120 ms, and PRc interval ≥ 200 ms and PR < 200 ms (Table 3). Dyssynchrony data were demonstrated based on horizontal and vertical dyssynchrony, SPWMD, SLWSD, inter-VD, intra-VD, and DFTc (Table 4). Inter-VD and intra-VD data by Doppler, DTI, and color-coded DTI methods are presented in Table 5; however, the values regarding RV free wall delay based on the presence of peak of strain after pulmonic valve closure are denoted in Table 6).Classification of severity is also shown in Table 6. Correlation of severity of intra-VD and QRS duration and cross-tabulation of intra-VD according to QRSc limits is shown in Tables 7 and 8. Accordingly, 100% (8 out of 8) of DCM patients with prolonged QRS (QRSc ≥ 120 ms) and 83% (24/29) of patients with narrow QRS (QRSc < 120 ms) had intra-VD, in which both groups were differentiated based on the severity of dyssynchrony (Tables 7 and 8; Fig. 1).

**Table 7:** Correlation of severity of intra-VD with QRS duration

Severity of intra-VD	QRS limit		Total
	QRSc < 120	QRSc ≥ 120	
1	5, 17%	0	5
2	3, 10%	0	3
3	15, 51%	3, 37%	18
4	6, 13%	5, 63%	11
	29	8	37

Refer to list of abbreviations before references

Severity of dyssynchrony scoring 1–4 from no VD to severe VD, score 1 = no dyssynchrony: Score2 = mild, score 3 = mod, score 4: severe

**Table 8:** Cross-tabulation of correlation of QRS limit and intra-VD

		QRS limit		Total
		QRS ≥ 120 ms	QRS < 120 ms	
Intravent dyssynchrony	Nearly absent	0	5, 17%	5, 17%
	Present	8, 100%	24, 83%	32, 87%
		8, 22%	29, 78%	37

Refer to list of abbreviations before references

Correlation of severity of AVD and PRc interval and cross-tabulation of AVD and PRc interval are indicated in Tables 9 and 10. Based on our results, there were 16 out of 37 patients without AVD. Differentiation of their severity was also denoted for both group of PRc < 200 ms and PR ≥ 200 ms (Tables 9 and 10; Fig. 2). Data of vertical dyssynchrony for different segments between base and mid-portion are determined, which are notified in Table 4.

**Table 9:** Correlation of severity of AV dyssynchrony with PR interval

Severity of AVD	PRc limit		Total
	PRc < 200	PRc ≥ 200	
1	13, 54%	3, 23%	16
2	9, 36%	4, 30%	13
3	1, 4%	4, 30%	5
4	1, 4%	2, 8%	3
	24	13	37

Refer to list of abbreviations before references

Severity of AV dyssynchrony scoring 1–4 from no AVD through severe AVD (score 1 = no AVD, 2: 0.35 s < DFT ≤ 0.40 s, 3: 0.3 s < DFT ≤ 0.35 s, 4: DFT ≤ 0.3 s

**Table 10:** Cross-tabulation of correlation of PR limit and AVD

		PR limit		total
		PRc ≥ 200 ms	PRc < 200 ms	
AVD	Absent	3, 23%	13, 54%	16, 43%
	Present	10, 77%	11, 46%	21, 57%
		13, 35%	24, 65%	37

Refer to list of abbreviations before references

The similarity between tissue Doppler and color-coded DTI with strain analysis showed that SLWSD of 24% by DTI and 32% by color-coded DTI of which 88% and 83%, respectively, was among grades 3–4 intra-VD by strain scoring (Tables 5 and 6).

LVEF was lower and LV GLS, mortality, Pro-BNP, NYHA FC, and severity of intra-VD were higher in the group with QRS ≥ 120 ms and PR ≥ 200 ms. The effect size was in favor of valuable effect of prolonged QRS on worsening LV and RV GLS, LV and RV dyssynchrony score, LVEF, and pro-BNP. Likewise, it indicated the valuable effect of prolonged PR on deteriorating LVEF, LV GLS, LV dyssynchrony score, and pro-BNP. Also OR, effect size, and P value showed that prolonged QRS and PR could be accounted as some risk factors for mortality and clinical manifestation (Table 11).

**Table 11:** Some characteristics of patients with two different QRS and PR limits groups

Group	LVEF%	RVEF%	LV GLS	RV GLS	LV Dys score	RV Dys score	Mortality	Pro-BNP	NYHA median ± SD
	Mean ± SD	Mean ± SD	Mean ± SD	Mean ± SD	Mean ± SD	Mean ± SD	Mean ± SD	Mean ± SD	
QRS ≥ 120 (no = 8)	15% ± 9%	30% ± 20%	− 4% ± 3%	− 8% ± 4%	18.6 ± 4.5	1.5 ± 0.5 (1–2)	50%	13,947 ± 14,823	3 ± 0.7 Range = 2–4
QRS < 120 (no = 29)	21% ± 13%	28% ± 15%	− 7% ± 5%	− 10% ± 5%	13.8 ± 8.1	1.5 ± 2 (0–8)	20.7%	6818.5 ± 8064	2 ± 0.9 Range = 1–4
*P* value/ES/OR	*P* = 0.135 ES = 0.54	*P* = 0.912 ES = 0.06	*P* = 0.160 ES = 0.61	*P* = 0.149 ES = 0.52	*P* = 0.215 ES = 0.58	*P* = 0.119 ES = 0.67	*P* = 0.161 ES = 0.64	*P* = 0.098 OR = 3.83 CI = 0.95 (0.73–19.99)	*P* = 0.095 ES = 1.01
PR ≥ 200 (no = 13)	14% ± 6%	27% ± 13%	− 4% ± 3%	− 8% ± 3%	18.8 ± 5.2	1.07 ± 0.8	61.5%	14,109 ± 12,082	3 ± 0.76 Range = 2–4
PR < 200 (no = 24)	22% ± 14%	30% ± 18%	− 8% ± 5%	− 10% ± 6%	12.7 ± 8	1.75 ± 2.1	8.3%	5245 ± 7411	2 ± 0.9 Range = 1–4
P value/ES/OR	*P* = 0.088 ES = 0.80	*P* = 0.86 ES = 0.19	*P* = 0.044* ES = 0.87	*P* = 0.175 ES = 0.10	*P* = 0.014* ES = 0.98	*P* = 0.570 ES = 0.02	*P* = 0.026* ES = 0.91	*P* = 0.001* OR = 17.6 CI = 0.95 (2.82–109.56	*P* = 0.077 ES = 1

Refer to list of abbreviations before references

ES, effect size; OR, odds ratio, *shows significant *P* value (NS at *P* > 0.05)

The evaluation of severity of dyssynchrony of different segments indicated that inferolateral, anterolateral, anterior, anteroseptal walls were among the most severe delayed segments sequentially (Table 12).

**Table 12:** Severity of dyssynchrony at different segments

Segments	Mild	Mod	Severe/paradox	Total score
Inferolateral	6	8	38	174
Anteroseptal	11	7	11	69
Inferoseptal	25	4	8	65
Anterolateral	16	26	12	116
Anterior	9	13	10	75
Inferior	7	9	1	29
RV free wall	26	0	0	26

Refer to list of abbreviations before references

Most dyssynchrony parameters were correlated appropriately with ventricular function and ECG parameters. Gender was not correlated with any parameters. Although fibrosis correlation with mortality, GLS, and LVEF was not significant, it was significantly positive with age, LBBB, and QRS interval (Tables 13 and 14).

**Table 13:** Some correlation of dyssynchrony with echo and electrocardiographic parameters

	LVEF	RVEF	LV GLS	RV GLS	LVEDV	LVESV	RVEDV	RVESV	QRSc	PRc
Intra-VD (strain)	R = − 0.80	R = − 0.55	R = 0.74	R = 0.64	R = 0.58	R = 0.66	R = 0.39	R = 0.51	R = 0.38	R = 0.61
	P = 0.000	P = 0.000	P = 0.000	P = 0.000	P = 0.000	P = 0.000	P = 0.015	P = 0.001	P = 0.019	P = 0.000
DFTc	R = 0.71	R = 0.48	R = − 0.64	R = − 0.40	R = − 0.46	R = − 0.52	R = − 0.16	R = − 0.27	R = − 0.49	R = − 0.76
	P = 0.000	P = 0.002	P = 0.000	P = 0.013	P = 0.004	P = 0.001	P = 0.32	P = 0.10	P = 0.002	P = 0.000
Inter-VD, dop	R = − 0.40	R = − 0.10	R = 0.42	R = 0.21	R = 0.38	R = 0.39	R = − 0.039	R = − 0.77	R = 0.51	R = 0.58
	P = 0.013	P = 0.55	P = 0.008	P = 0.20	P = 0.019	P = 0.015	P = 0.81	P = 0.65	P = 0.001	P = 0.000
Inter-VD,DTI	R = − 0.39	R = − 0.12	R = 0.39	R = 0.29	R = 0.38	R = 0.39	R = − 0.12	R = − 0.058	R = 0.47	R = 0.49
	P = 0.015	P = 0.47	P = 0.017	P = 0.075	P = 0.020	P = 0.016	P = 0.44	P = 0.73	P = 0.003	P = 0.002
Inter-VD,CC	R = − 0.31 P = 0.06	R = − 0.17 P = 0.29	R = 0.34 P = 0.03	R = 0.33 P = 0.05	R = 0.24 P = 0.13	R = 0.26 P = 0.11	R = − 0.23 P = 0.17	R = − 0.13 P = 0.44	R = 0.43 P = 0.007	R = 0.50 P = 0.001
Inter-VD,strain	R = 0.45	R = 0.46	R = 0.49	R = 0.57	R = 0.25	R = 0.32	R = 0.21	R = 0.37	R = 0.044	R = 0.10
	P = 0.005	P = 0.004	P = 0.002	P = 0.000	P = 0.125	P = 0.052	P = 0.199	P = 0.021	P = 0.79	P = 0.54
SPWMD	R = − 0.47	R = − 0.22	R = 0.53	R = 0.41	R = 0.34	R = 0.38	R = 0.11	R = 0.09	R = 0.60	R = 0.58
	P = 0.003	P = 0.17	P = 0.001	P = 0.03	P = 0.03	P = 0.02	P = 0.5	P = 0.58	P = 0.000	P = 0.000
SLWSD DTI	R = − 0.49	R = − 0.25	R = 0.48	R = 0.48	R = 0.57	R = 0.59	R = 0.34	R = 0.36	R = 0.33	R = 0.38
	P = 0.002	P = 0.12	P = 0.002	P = 0.002	P = 0.000	P = 0.000	P = 0.04	P = 0.03	P = 0.04	P = 0.02

Refer to list of abbreviations before references, significant *P* value at P ≤ 0.05

**Table 14:** Correlation of dyssynchrony with other parameters

	Mortality	Age	Sex	Pro-BNP	FC	SLWMD DTI	LGE fibrosis	LBBB	QRS dur
Intra-VD (strain)	R = 0.37 P = 0.02	R = 0.03 P = 0.84	R = − 0.12 P = 0.46	R = 0.48 P = 0.002	R = − 0.69 P = 0.000	R = 0.82 P = 0.000	R = 0.2 P = 0.2	R = 0.17 P = 0.30	R = 0.38 P = 0.019
DFTc	R = − 0.30 P = 0.067	R = − 0.01 P = 0.95	R = − 0.16 P = 0.33	R = − 0.37 P = 0.024	R = − 0.50 P = 0.002	R = − 0.23 P = 0.16	R = − 0.25 P = 0.16	R = − 0.17 P = 0.30	R = − 0.49 P = 0.002
Inter-VD, dop	R = 0.56 P = 0.000	R = 0.036 P = 0.83	R = 0.23 P = 0.15	R = 0.44 P = 0.006	R = 0.48 P = 0.002	R = 0.17 P = 0.3	R = 0.2 P = 0.25	R = 0.37 P = 0.024	R = 0.51 P = 0.01
Inter-VD, DTI	R = 0.37 P = 0.02	R = − 0.004 P = 0.98	R = 0.19 P = 0.24	R = 0.39 P = 0.01	R = 0.19 P = 0.25	R = 0.18 P = 0.27	R = 0.16 P = 0.36	R = 0.3 P = 0.07	R = 0.47 P = 0.03
Inter-VD, CC	R = 0.42 P = 0.008	R = 0.198 P = 0.24	R = 0.29 P = 0.08	R = 0.29 P = 0.07	R = 0.38 P = 0.02	R = 0.3 P = 0.06	R = 0.22 P = 0.22	R = o.22 P = 0.18	R = 0.44 P = 0.007
Inter-VD, strain	R = 0.26 P = 0.11	R = 0.06 P = 0.71	R = 0.03 P = 0.84	R = 0.35 P = 0.03	R = 0.50 P = 0.002	R = 0.1 P = 0.5	R = 0.38 P = 0.03	R = 0.18 P = 0.29	R = 0.37 P = 0.024
SPWMD	R = 0.35 P = 0.03	R = 0.20 P = 0.21	R = − 0.004 P = 0.98	R = 0.22 P = 0.17	R = 0.50 P = 0.002	R = 1	R = 0.25 P = 0.15	R = 0.30 P = 0.06	R = 0.34 P = 0.04
LGE fibrosis	R = 0.1 P = 0.5	R = 0.45 P = 0.009	R = 0.04 P = 0.8	R = 0.27 P = 0.12	R = 0.28 P = 0.11	R = 0.25 P = 0.15	R = 1	R = 0.49 P = 0.004	R = 0.36 P = 0.04

Refer to list of abbreviations before references, significant P value at P ≤ 0.05

Our study showed 27% inter-VD by spectral DTI method and 38% by Doppler method where differentiation by QRSc was in favor of lack of complete correspondence of electrical and mechanical dyssynchrony (Tables 15 and 16).

**Table 15:** Cross-tabulation of AO-PA diff Doppler with QRS interval

		QRS ≥ 120 ms	QRS < 120 ms	Total
Inter-VD	Present	6, 75%	8, 27%	14, 38%
	Absent	2, 25%	21, 73%	23, 62%
		8	29	37

Refer to list of abbreviations before references

**Table 16:** Cross-tabulation of AO-PA diff DTI Doppler with QRS interval

		QRS ≥ 120 ms	QRS < 120 ms	Total
Inter-VD	Present	3, 37%	7, 24%	10, 27%
	Absent	5, 63%	22, 76%	27, 73%
		8	29	37

Refer to list of abbreviations before references

## Discussion

Numerous studies have highlighted the role of mechanical dyssynchrony in adult patients with heart failure, primarily to ameliorate patient selection for CRT. Our objective was to clarify different types of dyssynchrony to facilitate the application of CRT in pediatric patients.

Our study showed that 100% of DCM patients with prolonged QRS (QRS ≥ 120 ms) had intra-VD, of which 12.5% had mild, 25% mod and 62.5% severe dyssynchrony. However, around 83% of patients with narrow QRS (QRS < 120 ms) had intra-VD, of which 10% were mild, 52% mod, and 21% severe dyssynchrony and also revealed that 77% of DCM patients with prolonged PRc had AVD of which around 31% were mild, 31% mod, and 15% severe AVD, while among PRc < 200 ms, 54% did not have AVD, 38% mild AVD, 4% mod, and another 4% had severe AVD. Moreover, our study showed the presence of remarkable effect of prolonged QRS and PR on worsening LV and RV GLS, LV, and RV dyssynchrony score, LVEF, and pro-BNP. Likewise, they both could be accounted as some risk factors for mortality and clinical manifestation.

In the presence of prolonged PR interval, LV PET, IVCT, and IVRT are prolonged and ET is of short duration, actually interpreting as much wasted time and intrinsic workload without blood displacement, so MV opening is delayed and DFT is decreased, in which the correspondence was also demonstrated in our study [5, 10]. AVD can result in impaired filling of the LV, resulting in diminished stretching of myocyte and reduced LV stroke volume via the Frank–Starling mechanism; also concomitant contraction of the LA and the LV can induce retrograde blood flow into the pulmonary veins and, possibly, pulmonary edema [6, 7].

Classical pattern of dyssynchrony, as described by Forsha et al., is characterized by the early contraction of at least one septal segment and early stretching in at least one opposing lateral wall segment. This pattern is further defined by the presence of late post-systolic peak contraction occurring after aortic valve closure in the segment that initially experienced early stretching. The classical pattern was demonstrated in 38% of subjects with 2nd and 3rd AVB, all of whom had LBBB and LV dysfunction and also 35% of whom had elongated time to peak of ejection where seen in only 2% of non-classical pattern subjects [11]. They had previously performed a similar work on DCM pediatric patients for presence of classical pattern dyssynchrony, of which 85% of them had wide QRS [12]. Those findings chime with our study results regarding the compatibility of severity of dyssynchrony based on strain and tissue imaging (Tables 5 and 6) and also the correlation of severity of intra-VD and QRS interval (Tables 11 and 13).

In a study involving patients with advanced heart failure, TDI revealed that 27% of patients with a narrow QRS complex (< 120 ms) exhibited mechanical dyssynchrony. Conversely, 30% of patients with a very wide QRS complex (> 150 ms) showed no significant mechanical dyssynchrony [6]. Contrastingly, our study utililizing strain analysis found that 100% of DCM patients with prolonged QRS (QRSc ≥ 120 ms) had intra-VD, and 83% of patients with narrow QRS (QRSc < 120 ms) also exhibited intra-VD. This finding was determined by the later occurrence of peak strain after aortic valve closure time. Importantly, the severity of dyssynchrony in our patients ranged from mild to severe based on the scoring measurements, a finding that was not reported in the aforementioned study [6]. Moreover, our study showed the presence of remarkable effect of prolonged QRS and PR on worsening LV and RV GLS, LV, and RV dyssynchrony score, LVEF, and pro-BNP. Likewise, they both could be accounted as some risk factors for mortality and clinical manifestation (Table 11).

According to some research, a difference of more than 40 ms between the two velocities on CC DTI is considered abnormal [5, 6]. Thereby, our study showed 27% inter-VD by DTI Doppler method, of which 30% were among patients with prolonged QRS and 70% of narrow QRS group; however, the time difference was adjusted by HR in our patients. All of these data are in favor of lack of complete correspondence of electrical and mechanical dyssynchrony (Tables 15 and 16). We obtained inter-VD by other methods too, in which their cross-tabulation results waived in this article but are available on request. Based on strain analysis, we found no delay in 30% and just mild delay in 70%.

In earlier research, pre-CRT longitudinal strain analysis revealed a typical LBBB contraction pattern characterized by the presence of a septal flash (a rapid, short inward motion of the septum) and apical rocking (a transverse motion of the apex during systole in both left and right directions). These patterns demonstrated the most convincing evidence for clinical application. Post-CRT implantation, persistent or worsening dyssynchrony was directly correlated with an increased risk of fatal arrhythmias [13, 14]. Nevertheless, our patients were more likely candidates for heart transplantation rather than CRT, primarily due to the rare occurrence of actual LBBB in this cohort.

SPWMD has been suggested as a method to assess dyssynchrony in patients with heart failure, with a cutoff value of more than 130 ms between 2 opposing segments. Still, the cutoff point is not well defined in the pediatric age group due to higher and more variable heart rates. Moreover, the low reproducibility of SPWMD has been noted in previous studies, which limits its clinical value and practical application [6, 7]. However, in our study, only 37% of patients had SPWMD exceeding 130 ms, significantly lower than the 86% obtained from strain analysis. This discrepancy could primarily be attributed to the higher heart rate in children, suggesting a lower cutoff value may be more appropriate for identifying SPWMD in this population. Our findings are consistent with a study by Cazeau et al., which concluded that no single electromechanical parameter could fully describe mechanical dyssynchrony [14]. Additionally, a decrease in left time to ejection by TDI was found to be a favorable predictor of resynchronization, while an increase in DFT and a decrease in SPWMD were not considered good CRT targets [15].

Intra-VD can be evaluated using TDI and strain analysis, both of which provide insight into myocardial motion. Motion can be represented as velocity, displacement, or strain. In a healthy heart, the peaks of motion for all segments occur simultaneously, resulting in velocity curves from all regions of the LV peaking together during early systole, while strain curves peak at the end of systole. Conversely, in a dyssynchronous heart, the peaks of regional wall motion curves do not overlap [7]. However, detecting peak velocity can be challenging in some patients due to the presence of multiple, jagged, or flat peaks in the TDI color-coded velocity curves. Further, the timing of peak velocities can be significantly affected by even small changes in the placement of the region of interest. Additionally, TDI is unable to differentiate between active contraction and passive motion resulting from tethering or translational movement of the heart, which may further complicate the interpretation of results [6, 7]. A cutoff value of 65 ms has been suggested [6] which accordingly, our study showed the presence of 29% inter-VD by CC DTI peak values (Table 5).

We utilized strain analysis for assessing intra-VD since strain offers advantages in distinguishing motion resulting from active contraction, as opposed to tethering or translational motion of the heart. This analysis enables more accurate identification of delays for each segment. In addition, considering the higher and more variable heart rate in children and the lower reproducibility associated with TDI, strain analysis emerges as a more reliable and precise method for assessing intra-VD in this population.

LBBB is observed in approximately 30% of patients with heart failure [6]. Nonetheless, we found this pattern in only 10.8% of our patients, which could be explained by the age group difference.

Some investigators found that a time difference of > 130 ms between peak septal wall and posterior wall radial strain predicted long-term response to CRT [6]. Given the higher and more variable heart rate in children, the time difference required for the designation of dyssynchrony may be lower than 130 ms. To account for this, we partitioned the delay following aortic valve closure into three segments: the first, second, and third segments, which we termed mild, moderate, and severe (paradoxical) dyssynchrony, respectively. We then tallied the number of segments exhibiting mild, moderate, and severe dyssynchrony, as detailed in the Methods section.

Andrei et al. demonstrated that cardiac electromechanical dyssynchrony could be improved by hemodynamic treatment, including fluid expansion and inotropic drugs, in adult patients with hemodynamic instability. Their study established a connection between dyssynchrony and ventricular–arterial coupling [16].

A study involving heart failure patients with narrow QRS complexes revealed a significant electromechanical delay during peak physical exercise [17]. Another study in pediatric patients with DCM demonstrated that more severe LV discoordination was linked to increased LV dysfunction and a higher likelihood of death or transplantation [18].

A recent study in children with pulmonary hypertension revealed that RV and LV mechanical dyssynchrony, along with RV dilatation, were prognostic factors for poorer clinical outcomes [19]. Our study’s findings, demonstrating a direct correlation between mortality rate, functional capacity, and pro-BNP levels and the severity of intra-VD, inter-VD, and AVD, further support these observations (Table 14).

Using CMR, a study examining fibrosis in patients with heart failure revealed a link between greater degrees of intra-VD and fibrosis [20]. Our findings, however, indicated a nonsignificant positive correlation between the presence of fibrosis, as shown by late gadolinium enhancement in CMR, and both intra-VD and mortality. This discrepancy might be attributed to the fact that CMR was not performed for 5 of our patients, and our study’s follow-up period for mortality was both short and heterogeneous. While the correlation between fibrosis and mortality, GLS, and LVEF was nonsignificant, there was a significant positive correlation with age, LBBB, and QRS duration.

In a multivariate analysis including guideline criteria for CRT (i.e., QRS width, presence of LBBB, and ejection fraction), interventricular mechanical delay and Tei index, baseline DFT was the strongest predictor of adverse outcome [10].

Equilibrium radionuclide angiography (ERNA) has been proposed as a method to assess the severity of dyssynchrony by examining the dynamic structure associated with QRS and PR intervals, as well as LVEF. This approach aims to differentiate between absent, mild, and moderate-to-severe inter-VD and intra-VD [21]. A prior study found that dyssynchrony of papillary muscles with a value greater than 30 ms was predictive of moderate-to-severe mitral regurgitation in DCM patients [22].

In our study, the highest dyssynchrony scores, calculated based on the time of appearance of peak systolic strain after aortic valve closure (Table 6), were associated with the inferolateral, anterolateral, anterior, and anteroseptal walls, in descending order. Overall, advancements in echocardiographic techniques have the potential to improve its accuracy compared with CMR. Furthermore, the affordability, accessibility, and applicability of echocardiography in unstable hospitalized patients make it a more suitable option for monitoring patients with DCM.

According to this study results, we suggest that the acquisition of dyssynchrony parameters by echocardiography including inter-VD, intra-VD, and AVD can be used to identify the optimal candidates for cardiac resynchronization therapy (CRT) to enable achieving maximum benefit from CRT. It is obvious that the application of CRT can improve the systolic and diastolic ventricular function and reduces functional mitral regurgitation to great extent; thereby, it would be useful to postpone the heart transplant, as it would be encountered the scarcity, the impediments and later potential complications.

## Conclusions

Our findings indicated that DCM causes both intra- and inter-VD, associated with QRS duration concerning severity, and also results in AVD that are correlated with PRc interval. Notably, a substantial proportion of patients with narrow QRSc also demonstrated intra-VD and inter-VD, while nearly half of those with normal PRc exhibited AVD. Collectively, these observations suggest a lack of complete correspondence between electrical and mechanical dyssynchrony.

### Limitations of the study

The major limitation of our study is the small number of subjects. The second is that we had to perform echocardiography by ICU echosystem of GE S 60—which did not have the ability of taking GCS and GRS for inward patients, that led to the absence of GCS in most subjects and GRS at all. Furthermore, because of the higher and more variable HR in children, the time difference needed for the assignment of dyssynchrony in children might be lower than 130 ms. Therefore, we divide the amount of delay after AVC into three parts: the first, the second, and the third segments, namely mild, mod, and severe/paradoxical dyssynchrony, respectively**.** The third was that our patients were non-homogenous, regarding the severity of systolic dysfunction (most of patients had severe systolic dysfunction). The next was the unequal follow-up periods to determine the death rate, given the longer follow-up period for the earlier subjects entered in the study and the shorter follow-up periods for the later ones. The other shortcoming was that, given the lack of 3D deformation data because of non-availability of relevant software, we had to detect strain analysis just in two directions, which may not optimally reflect 3D heart motion. Finally none of our patients were undertaken CRT, so our mechanical dyssynchrony data could not be checked prior to and following that. Therefore, the further studies would be suggested in this regard to increase the accuracy and applicability.

## Data Availability

All data are available upon request from the corresponding author.

## Abbreviations

AL: Anterolateral
Ant: Anterior
AS: Anteroseptal
AVC: Aortic valve closure
AVD: Atrioventricular dyssynchrony
AVO: Aortic valve opening
CC TDI: Color-coded tissue Doppler image
CHD: Congenital heart disease
CMR: Cardiac magnetic resonance imaging
CRT: Cardiac resynchronization therapy
DCM: Dilated cardiomyopathy
DFTc: Diastolic filling time corrected
EDV: End diastolic volume
EMD: Electromechanical delay
ESD: Electrosystolic delay
ESV: End systolic volume
ET: Ejection time
FC: Functional class
GCS: Global circumferential strain
GLS: Global longitudinal strain
GRS: Global radial strain
HF: Heart Failure
HR: Heart rate
IL: Inferolateral
Inf: Inferior
IS: Inferoseptal
IVCT: Isovolumic contraction time
IVRT: Isovolumic relaxation time
LBBB: Left bundle branch block
LGE: Late gadolinium enhancement
LVEF: Left ventricular ejection fraction
LVFT: Left ventricular filling time
LV PET: Left ventricular pre ejection time
MR: Mitral regurgitation
NCLV: Non-compaction LV
NT pro-BNP: N-terminal pro-B-type natriuretic peptide
PW TDI: Pulse-wave tissue Doppler images
PRc: PR interval corrected
PS: Peak of strain
PVC: Pulmonic valve closure
Pdx: Paradoxical
QRSc: QRS duration corrected
ROI: Region of interest
RVEF: Right ventricular ejection fraction
SPWMD: Septo post-wall mechanical delay
SLWSD: Septo lateral wall systolic delay
TR: Tricuspid regurgitation
VD: Ventricular delay

## References

[1] Albakri A (2018) Dilated cardiomyopathy: a review of literature on clinical status and meta-analysis of diagnostic and clinical management. J Clin Invest Stud. 10.15761/JCIS.1000107

[2] Ghader FR, Abaskhanian ZA (2009) Influence of metoprolol on systolic and diastolic function in children with heart failure. Pak J Biol Sci 12(5):451–454. 10.3923/pjbs.2009.451.454 19579987 10.3923/pjbs.2009.451.454

[3] Moradian M, Rashidighader F, Golchinnaghash F, Meraji M, Ghaemi HR (2023) Impact of pulmonary valve replacement on left and right ventricular function using strain analysis, in children with repaired tetralogy of Fallot. Egypt Heart J 75(1):51. 10.1186/s43044-023-00379-w 37335364 10.1186/s43044-023-00379-w PMC10279607

[4] Matsumoto K, Tanaka H, Miyoshi T, Hiraishi M, Kaneko A, Fukuda Y, Tatsumi K, Kawai H, Hirata K (2013) Dynamic left ventricular dyssynchrony assessed on 3-dimensional speckle-tracking area strain during dobutamine stress has a negative impact on cardiovascular events in patients with idiopathic dilated cardiomyopathy. Circ J 77:1750–1759. 10.1253/circj.cj-12-1487 23558740 10.1253/circj.cj-12-1487

[5] Anzouan-Kacou JB, Ncho-Mottoh MP, Konin C, N’guetta AR, Ekou KA, Koffi BJ, Soya KE, Tano ME, Abouo-N’dori R (2012) Prevalence of cardiac dyssynchrony and correlation with atrio-ventricular block and QRS width in dilated cardiomyopathy: an echocardiographic study. Cardiovasc J Afr 23(7):385–388. 10.5830/CVJA-2012-032 22914996 10.5830/CVJA-2012-032 PMC3721890

[6] Bank AJ, Burns KV, Gage RM (2010) Echocardiographic measurement of mechanical dyssynchrony in heart failure and cardiac resynchronization therapy. US Cardiol 7(1):24–32. 10.15420/usc.2010.7.1.24

[7] Arita T, Sorescu GP, Schuler BT, Schmarkey LS, Merlino JD (2007) Speckle-tracking strain echocardiography for detecting cardiac dyssynchrony in a canine model of dyssynchrony and heart failure. Am J Physiol Heart Circ 293:H735–H742. 10.1152/ajpheart.00168.2007 10.1152/ajpheart.00168.2007 17449554

[8] Faggiano A, Avallone C, Gentile D, Provenzale G, Toriello F, Merlo M, Sinagra G, Carugo S (2021) Echocardiographic advances in dilated cardiomyopathy. J Clin Med 10(23):5518. 10.3390/jcm10235518 34884220 10.3390/jcm10235518 PMC8658091

[9] Augustine DX, Coates-Bradshaw LD, Willis J, Harkness A, Ring L, Grapsa J et al (2018) Echocardiographic assessment of pulmonary hypertension: a guideline protocol from the British Society of Echocardiography. Echo Res Pract 5(3):11–24. 10.1530/ERP-17-0071 30012832 10.1530/ERP-17-0071 PMC6055509

[10] Verbrugge FH, Verhaert D, Grieten L, Dupont M, Rivero-Ayerza M, De Vusser P, Van Herendael H, Reyskens R, Vandervoort P, Tang WHW, Mullens W (2013) Revisiting diastolic filling time as mechanistic insight for response to cardiac resynchronization therapy. Europace 15:1747–1756. 10.1093/europace/eut130 23821473 10.1093/europace/eut130

[11] Forsha D, Gamboa DG, Risum N, Kropf PA, Hornik CP, Barker P, Kisslo J, Kanter RJ (2019) Electromechanical dyssynchrony and clinically silent ventricular dysfunction in young subjects with ventricular pacing for congenital and early acquired Av block. Biomed J Sci Tech Res 20:2. 10.26717/BJSTR.2019.20.003435

[12] Forsha D, Slorach C, Chen CK, Stephenson EA, Risum N, Hornik C, Wagner G, Mertens L, Barker P, Kisslo J, Friedberg MK (2014) Classic-pattern dyssynchrony and electrical activation delays in pediatric dilated cardiomyopathy. J Am Soc Echocardiogr 27(9):956–964. 10.1016/j.echo.2014.06.014 25063467 10.1016/j.echo.2014.06.014

[13] Satish P, Narasimhan B, Hagendorff A, Tayal B (2022) Evolving concept of dyssynchrony and its utility. J Geriatr Cardiol 19(1):44–51. 10.11909/j.issn.1671-5411.2022.01.010 35233222 10.11909/j.issn.1671-5411.2022.01.010 PMC8832040

[14] Stankovic I, Prinz C, Ciarka A, Daraban AM, Kotrc M, Aarones M, Szulik M, Winter S, Belmans A, Neskovic AN, Kukulski T, Aakhus S, Willems R, Fehske W, Penicka M, Faber L, Voigt J-U (2016) Relationship of visually assessed apical rocking and septal flash to response and long-term survival following cardiac resynchronization therapy (PREDICT-CRT). Eur Heart J Cardiovasc Imaging 17(3):262–269. 10.1093/ehjci/jev288 26588984 10.1093/ehjci/jev288

[15] Cazeau S, Toulemont M, Ritter P et al (2019) Statistical ranking of electromechanical dyssynchrony parameters for CRT. Open Heart 6:e000933. 10.1136/openhrt-2018-000933 30740229 10.1136/openhrt-2018-000933 PMC6347881

[16] Andrei S, Popescu BA, Caruso V, Nguyen M, Bouhemad B, Guinot PG (2022) Role of electromechanical dyssynchrony assessment during acute circulatory failure and its relation to ventriculo-arterial coupling. Front Cardiovasc Med 9:907891. 10.3389/fcvm.2022.907891 35800171 10.3389/fcvm.2022.907891 PMC9253504

[17] Dandrea A, Mele D, Nistri S, Riegler L, Galderisi M, Agricola E, Losi MA, Ballo P, Mondillo S, Badano LP (2013) Working Group Nucleus on Echocardiography of Italian Society of Cardiology. The prognostic impact of dynamic ventricular dyssynchrony in patients with idiopathic dilated cardiomyopathy and narrow QRS. Eur Heart J Cardiovasc Imaging 14(2):183–9. 10.1093/ehjci/jes154 22872513 10.1093/ehjci/jes154

[18] Forsha D, Slorach C, Chen CK, Sherman A, Mertens L, Barker P, Kisslo J, Friedberg MK (2016) Patterns of mechanical inefficiency in pediatric dilated cardiomyopathy and their relation to left ventricular function and clinical outcomes. J Am Soc Echocardiogr 29(3):226–236. 10.1016/j.echo.2015.11.011 26711366 10.1016/j.echo.2015.11.011

[19] Frank BS, Schäfer M, Douwes JM, Ivy DD, Abman SH, Davidson JA, Burzlaff S, Mitchell MB, Morgan GJ, Browne LP, Barker AJ, Truong U, von Alvensleben JC (2020) Novel measures of left ventricular electromechanical discoordination predict clinical outcomes in children with pulmonary arterial hypertension. Am J Physiol Heart Circ Physiol 318(2):H401–H412. 10.1152/ajpheart.00355.2019 31858817 10.1152/ajpheart.00355.2019 PMC7052618

[20] Lin LY, Wu CK, Juang JM, Wang YC, Su MY, Lai LP, Hwang JJ, Chiang FT, Tseng WY, Lin JL (2016) Myocardial regional interstitial fibrosis is associated with left intra-ventricular dyssynchrony in patients with heart failure: a cardiovascular magnetic resonance study. Sci Rep 6:20711. 10.1038/srep20711 26846306 10.1038/srep20711 PMC4742892

[21] Santos-Díaz A, Valdés-Cristerna R, Vallejo E, Hernández S, Jiménez-Ángeles L (2017) Automated classification of severity in cardiac dyssynchrony merging clinical data and mechanical descriptors. Comput Math Methods Med. 2017:3087407. 10.1155/2017/3087407 28348637 10.1155/2017/3087407 PMC5350313

[22] Tigen K, Karaahmet T, Dundar C, Guler A, Cevik C, Basaran O, Kirma C, Basaran Y (2010) The importance of papillary muscle dyssynchrony in predicting the severity of functional mitral regurgitation in patients with non-ischaemic dilated cardiomyopathy: a two-dimensional speckle-tracking echocardiography study. Eur J Echocardiogr 11(8):671–6. 10.1093/ejechocard/jeq040 20237053 10.1093/ejechocard/jeq040

